# Supplementary figures and images for: Comparison of feeding preferences of herbivorous fishes and the sea urchin Diadema antillarum in Little Cayman
Source: PeerJ. 2023 Nov 15;11:e16264. doi: 10.7717/peerj.16264 (PMC10656904; doi:10.7717/peerj.16264)

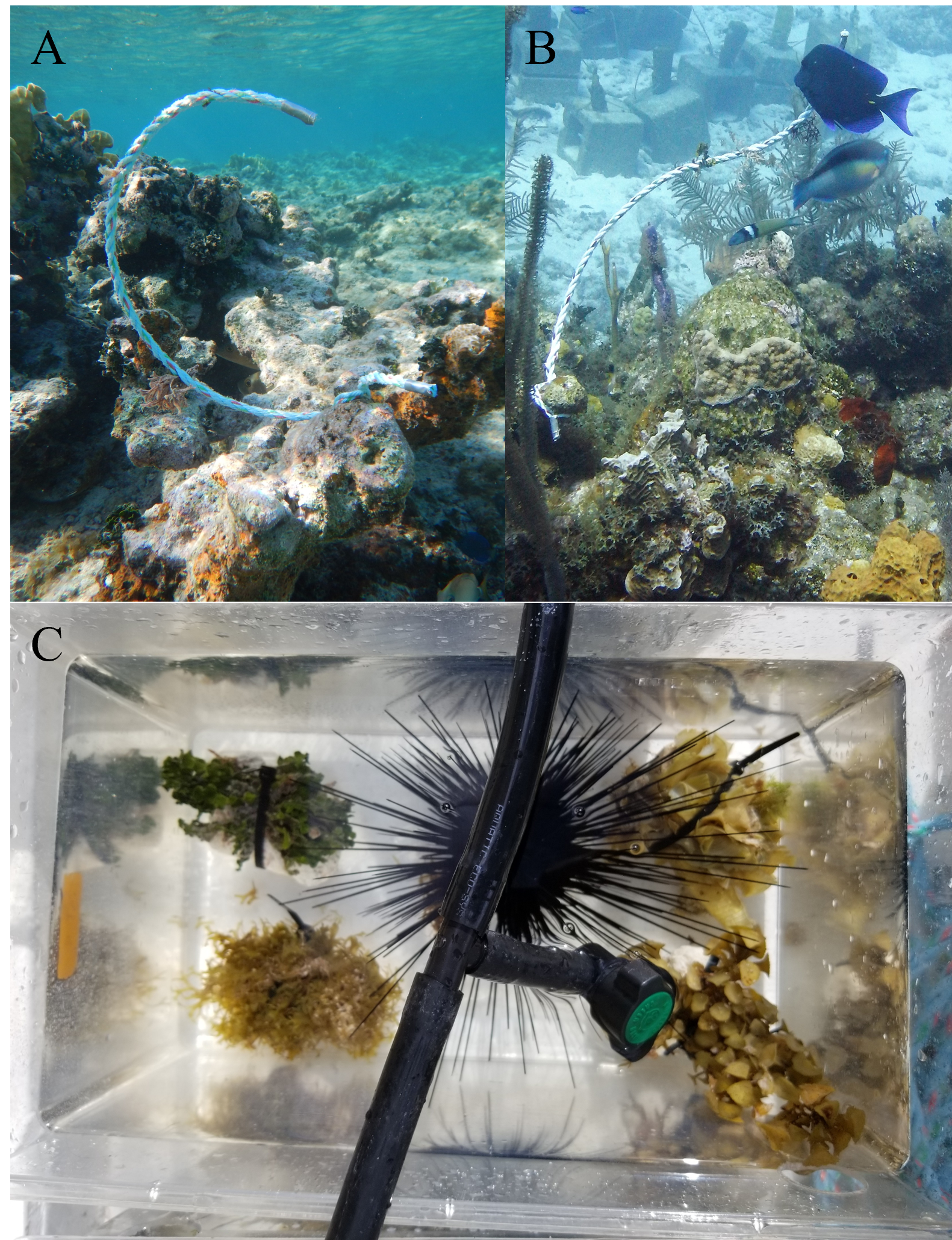

Supplement: Supplemental Information 1 [file peerj-11-16264-s001.png]
