# Supplementary material for: Comparison of feeding preferences of herbivorous fishes and the sea urchin Diadema antillarum in Little Cayman
Source: PeerJ. 2023 Nov 15;11:e16264. doi: 10.7717/peerj.16264 (PMC10656904; doi:10.7717/peerj.16264)
Supplement: Supplemental Information 2 — Percent eaten calculated for each macrophyte based on the average percent of lines with total consumption of macrophyte across all trials at the shallow (n = 7) and deep (n = 7) sites. [file peerj-11-16264-s002.docx]

| **Macrophyte** | **Herbivorous fishes-Shallow** | **Herbivorous fishes-Deep** |
| --- | --- | --- |
| *Dictyota* sp. | 24.44 ± 11.11 % | 4.44 ± 2.22 % |
| *Galaxaura* sp. | 0 | 81.67 ± 16.19 % |
| *Halimeda tuna* | 0 | 0 |
| *Laurencia* sp. 1 | 86.67 ± 3.85 % | 86.67 ± 10.18 % |
| *Laurencia* sp. 2 | 53.33 ± 3.85 % | 56.83 ± 5.78 % |
| *Lobophora* sp. | 96.67 ± 3.33 % | 95.56 ± 2.22 % |
| *Palisada* sp. | 68.89 ± 8.01% | 97.78 ± 2.22 % |
| *Thalassia testudinum* | 81.67 ± 7.39 % | 97.78 ± 2.22 % |
| *Turbinaria* sp. | 91.22 ± 8.89 % | 56.51 ± 17.05 % |
