# Supplementary material for: Comparison of feeding preferences of herbivorous fishes and the sea urchin Diadema antillarum in Little Cayman
Source: PeerJ. 2023 Nov 15;11:e16264. doi: 10.7717/peerj.16264 (PMC10656904; doi:10.7717/peerj.16264)
Supplement: Supplemental Information 3 — Generalized linear mixed models in R were used to assess the effect of the fixed factors, i.e., site and algae, on the proportion eaten while also accounting for the random factor of trial (glmmTMB(Eaten Algae*Site + (1—Trial), family = binomial), logit scale). Estimated marginal means were used to compare differences in the proportion eaten of each macrophyte within each site and compare the proportion eaten of each macrophyte between the shallow and deep sites. [file peerj-11-16264-s003.docx]

| **Model** | **AIC** | **logLIK** |
| --- | --- | --- |
| Eaten ~ Algae * Site + (1\|Trial) | 546.4 | -258.2 |
| **Random effects** | **Variance** | **SD** |
| Trial | 0.08688 | 0.2947 |

| ***Dictyota* sp.** | | | | | | | |
| --- | --- | --- | --- | --- | --- | --- | --- |
| Contrast | estimate | SE | df | t.ratio | p.value | Lower.CL | Upper.CL |
| Deep~Shallow | -1.466 | 0.731 | 642 | -2.006 | 0.045 | -2.901 | -0.031 |
| ***Laurencia* sp. 1** | | | | | | | |
| Contrast | estimate | SE | df | t.ratio | p.value | Lower.CL | Upper.CL |
| Deep~Shallow | -0.074 | 0.669 | 642 | -0.110 | 0.912 | -1.388 | 1.240 |
| ***Laurencia* sp. 2** | | | | | | | |
| Contrast | estimate | SE | df | t.ratio | p.value | Lower.CL | Upper.CL |
| Deep~Shallow | 0.236 | 0.499 | 642 | 0.473 | 0.637 | -0.744 | 1.216 |
| ***Lobophora* sp.** | | | | | | | |
| Contrast | estimate | SE | df | t.ratio | p.value | Lower.CL | Upper.CL |
| Deep~Shallow | -0.278 | 1.044 | 642 | -0.266 | 0.790 | -2.329 | 1.773 |
| ***Palisada* sp.** | | | | | | | |
| Contrast | estimate | SE | df | t.ratio | p.value | Lower.CL | Upper.CL |
| Deep~Shallow | 3.111 | 1.095 | 642 | 2.841 | 0.005 | 0.961 | 5.261 |
| ***Thalassia testudinum*** | | | | | | | |
| Contrast | estimate | SE | df | t.ratio | p.value | Lower.CL | Upper.CL |
| Deep~Shallow | 2.222 | 1.092 | 642 | 2.036 | 0.042 | 0.079 | 4.366 |
| ***Turbinaria* sp.** | | | | | | | |
| Contrast | estimate | SE | df | t.ratio | p.value | Lower.CL | Upper.CL |
| Deep~Shallow | -1.915 | 0.676 | 642 | -2.832 | 0.005 | -3.243 | -0.587 |

| **Deep** | | | | | | | |
| --- | --- | --- | --- | --- | --- | --- | --- |
| Contrast | estimate | SE | df | t.ratio | p.value | Lower.CL | Upper.CL |
| *Dictyota* sp. *~ Palisada* sp. | -6.574 | 1.200 | 642 | -5.480 | 1.27E-06 | -10.122 | -3.026 |
| *Dictyota* sp. *~ Laurencia* sp. 2 | -2.890 | 0.699 | 642 | -4.135 | 0.000792 | -4.957 | -0.823 |
| *Dictyota* sp. ~ *Lobophora* sp. | -5.765 | 0.959 | 642 | -6.012 | 6.41E-08 | -8.602 | -2.929 |
| Dictyota sp. ~ *Laurencia* sp. 1 | -4.580 | 0.770 | 642 | -5.949 | 9.26E-08 | -6.857 | -2.303 |
| Dictyota sp. ~ *Thalassia testudinum* | -6.399 | 1.182 | 642 | -5.413 | 1.83E-06 | -9.896 | -2.902 |
| Dictyota sp. ~ *Turbinaria* sp. | -2.912 | 0.701 | 642 | -4.152 | 0.001 | -4.986 | -0.837 |
| *Palisada* sp. ~ *Laurencia* sp. 2 | 3.684 | 1.085 | 642 | 3.395 | 0.013 | 0.474 | 6.893 |
| *Palisada* sp. *~ Lobophora* sp. | 0.808 | 1.260 | 642 | 0.642 | 0.995 | -2.918 | 4.535 |
| *Palisada* sp. ~ *Laurencia* sp. 1 | 1.994 | 1.120 | 642 | 1.781 | 0.562 | -1.318 | 5.305 |
| *Palisada* sp. ~ *T*. *testudinum* | 0.175 | 1.453 | 642 | 0.120 | 1.000 | -4.124 | 4.474 |
| *Palisada* sp. ~ *Turbinaria* sp. | 3.662 | 1.077 | 642 | 3.399 | 0.013 | 0.476 | 6.848 |
| *Laurencia* sp. 2 *~ Lobophora* sp. | -2.876 | 0.821 | 642 | -3.503 | 0.009 | -5.304 | -0.447 |
| *Laurencia* sp. 2 ~ *Laurencia* sp. 1 | -1.690 | 0.571 | 642 | -2.961 | 0.050 | -3.379 | -0.002 |
| *Laurencia* sp. 2 ~ *T*. *testudinum* | -3.509 | 1.082 | 642 | -3.242 | 0.021 | -6.711 | -0.307 |
| *Laurencia* sp. 2 ~ *Turbinaria* sp. | -0.022 | 0.459 | 642 | -0.048 | 1.000 | -1.380 | 1.336 |
| *Lobophora* sp. ~ *Laurencia* sp. 1 | 1.185 | 0.857 | 642 | 1.383 | 0.811 | -1.350 | 3.720 |
| *Lobophora* sp. ~ *T*. *testudinum* | -0.633 | 1.256 | 642 | -0.504 | 0.999 | -4.349 | 3.082 |
| *Lobophora* sp. ~ *Turbinaria* sp. | 2.854 | 0.806 | 642 | 3.539 | 0.008 | 0.468 | 5.239 |
| *Laurencia* sp. 1 ~ *T*. *testudinum* | -1.819 | 1.124 | 642 | -1.618 | 0.671 | -5.144 | 1.507 |
| *Laurencia* sp. 1 ~ *Turbinaria* sp. | 1.668 | 0.551 | 642 | 3.028 | 0.041 | 0.039 | 3.298 |
| *T*. *testudinum* ~ *Turbinaria* sp. | 3.487 | 1.085 | 642 | 3.215 | 0.023 | 0.279 | 6.695 |

| **Shallow** | | | | | | | |
| --- | --- | --- | --- | --- | --- | --- | --- |
| Contrast | estimate | SE | df | t.ratio | p.value | Lower.CL | Upper.CL |
| *Dictyota* sp. ~ *Palisada* sp. | -1.997 | 0.496 | 642 | -4.026 | 0.001 | -3.463 | -0.530 |
| *Dictyota* sp. ~ *Laurencia* sp. 2 | -1.188 | 0.496 | 642 | -2.395 | 0.202 | -2.655 | 0.279 |
| *Dictyota* sp. ~ *Lobophora* sp. | -4.577 | 0.818 | 642 | -5.597 | 6.76E-07 | -6.997 | -2.158 |
| Dictyota sp. ~ *Laurencia* sp. 1 | -3.188 | 0.627 | 642 | -5.087 | 9.91E-06 | -5.042 | -1.334 |
| Dictyota sp. ~ *Thalassia testudinum* | -2.710 | 0.535 | 642 | -5.071 | 1.07E-05 | -4.291 | -1.129 |
| Dictyota sp. ~ *Turbinaria* sp. | -3.361 | 0.646 | 642 | -5.205 | 5.43E-06 | -5.271 | -1.451 |
| *Palisada* sp. ~ *Laurencia* sp. 2 | 0.809 | 0.480 | 642 | 1.686 | 0.626 | -0.610 | 2.228 |
| *Palisada* sp. *~ Lobophora* sp. | -2.581 | 0.806 | 642 | -3.200 | 0.024 | -4.966 | -0.196 |
| *Palisada* sp. ~ *Laurencia* sp. 1 | -1.191 | 0.591 | 642 | -2.016 | 0.405 | -2.939 | 0.557 |
| *Palisada* sp. ~ *T*. *testudinum* | -0.714 | 0.496 | 642 | -1.439 | 0.780 | -2.181 | 0.754 |
| *Palisada* sp. ~ *Turbinaria* sp. | -1.365 | 0.658 | 642 | -2.075 | 0.369 | -3.310 | 0.580 |
| *Laurencia* sp. 2 *~ Lobophora* sp. | -3.390 | 0.821 | 642 | -4.131 | 0.001 | -5.816 | -0.963 |
| *Laurencia* sp. 2 ~ *Laurencia* sp. 1 | -2.000 | 0.641 | 642 | -3.121 | 0.031 | -3.896 | -0.104 |
| *Laurencia* sp. 2 ~ *T*. *testudinum* | -1.523 | 0.508 | 642 | -2.999 | 0.044 | -3.024 | -0.021 |
| *Laurencia* sp. 2 ~ *Turbinaria* sp. | -2.173 | 0.619 | 642 | -3.510 | 0.009 | -4.005 | -0.342 |
| *Lobophora* sp. ~ *Laurencia* sp. 1 | 1.390 | 0.858 | 642 | 1.619 | 0.670 | -1.149 | 3.928 |
| *Lobophora* sp. ~ *T*. *testudinum* | 1.867 | 0.805 | 642 | 2.318 | 0.237 | -0.515 | 4.249 |
| *Lobophora* sp. ~ *Turbinaria* sp. | 1.216 | 0.923 | 642 | 1.317 | 0.844 | -1.514 | 3.947 |
| *Laurencia* sp. 1 ~ *T*. *testudinum* | 0.477 | 0.574 | 642 | 0.831 | 0.982 | -1.222 | 2.177 |
| *Laurencia* sp. 1 ~ *Turbinaria* sp. | -0.173 | 0.777 | 642 | -0.223 | 1.000 | -2.472 | 2.125 |
| *T*. *testudinum* ~ *Turbinaria* sp. | -0.651 | 0.678 | 642 | -0.959 | 0.962 | -2.657 | 1.356 |
