# Supplementary material for: Comparison of feeding preferences of herbivorous fishes and the sea urchin Diadema antillarum in Little Cayman
Source: PeerJ. 2023 Nov 15;11:e16264. doi: 10.7717/peerj.16264 (PMC10656904; doi:10.7717/peerj.16264)
Supplement: Supplemental Information 4 — Bite numbers obtained from video recordings of the assays. Bite numbers were averaged across all trials for each herbivorous fish for each macrophyte and standardized to per hour. [file peerj-11-16264-s004.docx]

| Species | *Dictyota* sp. | *Galaxaura* sp. | *Halimeda tuna* | *Palisada* sp. | *Laurencia* sp. 1 | *Laurencia* sp. 2 | *Lobophora* sp. | *Thalassia testudinum* | *Turbinaria* sp. |
| --- | --- | --- | --- | --- | --- | --- | --- | --- | --- |
| **Shallow** | | | | | | | | | |
| *Acanthurus* spp. | 9.80 ± 5.03 | 5.40 ± 1.81 | 0 | 105.27 ± 22.94 | 50.79 ± 11.12 | 39.76 ± 11.00 | 0.83 ± 0.04 | 1.63 ± 0.71 | 6.56 ± 0 |
| *Sparisoma viride* (I) | 0 | 0 | 0 | 1.33 ± 0.55 | 0 | 0 | 0 | 18.75 ± 0 | 0 |
| *Sparisoma rubripinne* (I) | 1.88 ± 0 | 3.04 ± 0 | 0 | 5.63 ± 0 | 0 | 0 | 23.84 ± 23.12 | 8.59 ± 2.53 | 13.04 ± 0 |
| *Haemulon sciurus* | 0 | 1.56 ± 0 | 0 | 0 | 0 | 0 | 0 | 0 | 0 |
| *Kyphosus* sp. | 0 | 0 | 0 | 0 | 1.33 ± 0.54 | 0 | 0 | 0 | 0 |
| **Deep** | | | | | | | | | |
| *Acanthurus* spp. | 0 | 15.20 ± 6.35 | 1.07 ± 0 | 16.19 ± 10.99 | 50.67 ± 29.11 | 14.94 ± 12.20 | 7.14 ± 0 | 2.74 ± 0.48 | 7.50 ± 0 |
| *Thalassoma bifasciatum* | 0 | 10.59 ± 0 | 0 | 0 | 0 | 0 | 0 | 0 | 2.79 ± 0 |
| *Sparisoma aurofrenatum* (I) | 3.53 ± 0 | 0 | 0 | 0 | 6.79 ± 0 | 0 | 7.06 ± 0 | 24.46 ± 13.97 | 23.35 ± 21.06 |
| *Sparisoma aurofrenatum* (T) | 0 | 0 | 2.07 ± 0 | 0 | 0 | 0 | 0 | 6.21 ± 0 | 0 |
| *Sparisoma rubripinne* (I) | 4.71 ± 0 | 9.41 ± 0 | 0 | 0 | 0 | 0 | 4.71 ± 0 | 15.29 ± 0 | 0 |
| *Melichthys niger* | 0 | 0 | 0 | 32.07 ± 0 | 2.07 ± 0 | 0 | 0 | 0 | 0 |
